# Association of Socioeconomic and Geographic Factors With Diet Quality in US Adults

**DOI:** 10.1001/jamanetworkopen.2022.16406

**Published:** 2022-06-09

**Authors:** Marjorie L. McCullough, Sicha Chantaprasopsuk, Farhad Islami, Erika Rees-Punia, Caroline Y. Um, Ying Wang, Corinne R. Leach, Kristen R. Sullivan, Alpa V. Patel

**Affiliations:** 1 Department of Population Science, American Cancer Society, Kennesaw, Georgia; 2 Department of Surveillance and Health Equity Science, American Cancer Society, Kennesaw, Georgia

## Abstract

**Importance:**

Poor diet quality is a key factor associated with obesity and chronic disease. Understanding associations of socioeconomic and geographic factors with diet quality can inform public health and policy efforts for advancing health equity.

**Objective:**

To identify socioeconomic and geographic factors associated with diet quality in a large US cohort study.

**Design, Setting, and Participants:**

This cross-sectional study included adult men and women who enrolled in the Cancer Prevention Study-3 at American Cancer Society community events in 35 US states, the District of Columbia, and Puerto Rico between 2006 and 2013. Participants completed a validated food frequency questionnaire between 2015 and 2017. Data were analyzed from February to November 2021.

**Exposures:**

The main exposures included self-reported race and ethnicity, education, and household income. Geocoded addresses were used to classify urbanization level using Rural-Urban Commuting Area codes; US Department of Agriculture’s Food Access Research Atlas database classified residence in food desert.

**Main Outcomes and Measures:**

Poor diet quality was defined as lowest quartile of dietary concordance with the 2020 American Cancer Society recommendations for cancer prevention score, based on sex-specific intake categories of vegetables and legumes, whole fruits, whole grains, red and processed meat, highly processed foods and refined grains, and sugar-sweetened beverages.

**Results:**

Among 155 331 adults, 123 115 were women (79.3%), and the mean (SD) age was 52 (9.7) years), and there were 1408 American Indian or Alaskan Native individuals (0.9%); 2721 Asian, Native Hawaiian, or Pacific Islander individuals (1.8%); 3829 Black individuals (2.5%); 7967 Hispanic individuals (5.1%); and 138 166 White individuals (88.9%). All key exposures assessed were statistically significantly and independently associated with poor diet quality. Compared with White participants, Black participants had a 16% (95% CI, 8%-25%) higher risk of poor diet quality, while Hispanic/Latino had 16% (95% CI, 12%-21%) lower risk and Asian, Native Hawaiian, and Pacific Islander participants had 33% (95% CI, 26%-40%) lower risk of poor diet quality. After controlling for other characteristics, rural residence was associated with a 61% (95% CI, 48%-75%) higher risk of poor diet quality, and living in a food desert was associated with a 17% (95% CI, 12%-22%) higher risk. Associations of income with diet quality and education with diet quality varied by race and ethnicity (income: *P* for interaction = .01; education: *P* for interaction < .001). All diet score components were associated with disparities observed.

**Conclusions and Relevance:**

This cross-sectional study found that multiple individual-level socioeconomic and geographic variables were independently associated with poor diet quality among a large, racially and ethnically and geographically diverse US cohort. These findings could help to identify groups at highest risk of outcomes associated with poor diet to inform future approaches for advancing health equity.

## Key Points

**Question:**

Are social, economic, and geographic factors independently associated with diet quality?

**Findings:**

In this cross-sectional analysis of 155 331 adults participating in a nationwide US cohort study, Black individuals, White individuals with limited income, participants with low educational attainment, and people living in rural areas or food deserts were more likely to have overall poor diet quality. All dietary components, but especially sugar-sweetened beverages and processed meats, contributed to the disparities observed.

**Meaning:**

These findings suggest that multiple individual-level socioeconomic and neighborhood-level factors were independently associated with poor diet quality in this US cohort.

## Teaser

This cross-sectional study examines socioeconomic and geographic factors associated with diet quality in US adults.

## Introduction

Poor diet quality is associated with the leading causes of death in the US, including cancer.^1,2^ Diet quality tends to be lower among historically marginalized groups, such as American Indian and Alaska Native and Black communities, with roots in structural racism^3,4^ and among rural and economically disadvantaged communities that may have limited access to healthy food.^5,6^ These same communities have generally experienced disproportionately higher death rates of chronic diseases associated with obesity, in part owing to the association of diet quality with prevalence of obesity.^6^ A deeper understanding of how social, economic, and environmental factors affect diet quality could be helpful in addressing health disparities and advancing health equity.

For decades, numerous health organizations^7,8,9,10,11^ have published guidelines on healthy diet and lifestyle for prevention of cancer and other chronic diseases. A body of evidence suggests that greater dietary and behavioral concordance with the these guidelines is associated with significantly lower mortality from cancer, cardiovascular diseases, and all causes,^12,13,14^ including among people from historically marginalized racial and ethnic groups and individuals with limited resources.^15,16^ In the US, the proportion of cancers attributable to poor diet alone is estimated to be 4.2%^17^ to 5.2%^18^ and has recently been reported to vary substantially by race and ethnicity, education, and income.^19^ For example, in the US, non-Hispanic Black men and women were estimated to experience 214 more diet-associated cancer deaths per 100 000 population compared with non-Hispanic White individuals.^19^

However, the prevalence of obesity, poor diet, and physical inactivity in the US remains high. Two-thirds of US adults have overweight or obesity, and rates continue to increase.^20^ In the US and other high-income Western countries, the proportionate increase in body mass index (BMI; calculated as weight in kilograms divided by height in meters squared) over 30 years is greater in rural vs urban areas.^21^ Intakes of fruits, vegetables, and whole grains in the US are far below recommended intakes,^22,23^ and processed meat and added sugar intakes are above^24^ recommended intakes. Ultraprocessed foods, high in sugar, fat, and salt, contribute almost 60% of calories and 90% of added sugars in US diets.^25^ While trends in overall diet quality have improved in the US between 1999 and 2012 across race and ethnicity, education, and income groups, disparities in those improvements widened over time.^5^ Analyses that simultaneously evaluate socioeconomic and geographic factors associated with diet quality, and their interactions, are lacking.

The goal of this cross-sectional study was to identify socioeconomic and geographic factors associated with lower diet quality^7^ in the American Cancer Society’s (ACS) Cancer Prevention Study-3 (CPS-3),^26^ a large and diverse prospective cohort of US adults. Findings may reveal population subgroups that could benefit from targeted efforts to improve dietary behaviors for chronic disease prevention.

## Methods

The CPS-3 study protocol was approved by the institutional review board of Emory University. All participance provided written informed consent. This study follows the Strengthening the Reporting of Observational Studies in Epidemiology (STROBE) reporting guideline for observational studies.

### Study Population

Approximately 304 000 US adults aged 30 to 65 years with no history of cancer from 35 states, the District of Columbia, and Puerto Rico–which together account for 93% of the US population—voluntarily enrolled in CPS-3 between 2006 and 2013.^26^ The study was not designed to be nationally representative but to include participants with geographic and racial and ethnic diversity.^26^ During in-person enrollment at ACS fundraising events (eg, Relay for Life) or community enrollment drives designed for recruitment, participants completed a survey with questions on race and ethnicity and highest level of education attained, and provided written informed consent.^26^ After enrollment, participants completed a comprehensive baseline survey at home. Individuals who returned the baseline survey were recontacted every 3 years (ie, the active cohort) to update medical, demographic, and lifestyle information (254 650 individuals). Diet was assessed on the 2015 follow-up survey returned by 186 638 participants between 2015 and 2017; 177 345 participants (69.6% of the active cohort, including 36 419 men and 140 926 women) completed the food frequency questionnaire (FFQ).

### Diet Assessment

The CPS-3 FFQ was modified from the Willett/Harvard FFQ^27,28,29^ using 24-hour recalls and focus groups among Hispanic/Latino, non-Hispanic/Latino Black and non-Hispanic/Latino White, participants in CPS-3.^30^ It included 191 line items listing standard portion sizes and queried usual consumption over the past year with frequency ranging from never or fewer than 1 time per month to 4 times per day or more for foods and 6 times per day or more for beverages. The FFQ was validated for assessment of foods^31^ and nutrients,^30^ overall and within racial and ethnic groups and by education. The CPS-3 diet validation study was conducted in English, and less than 1% of Hispanic/Latino participants completed the Spanish language FFQ.

### Outcome: Dietary Quality

The ACS diet score reflects dietary recommendations of the 2020 ACS Guideline on Diet and Physical Activity for Cancer Prevention,^7^ which are consistent with recommendations for prevention of other chronic diseases^9,11^ (eTable 1 in the Supplement). Sex-specific consumption quartiles were used in scoring all components except for sugar-sweetened beverages (SSBs). Greater consumption quartile of vegetables and legumes, whole fruits, and variety of both (contributing 0-3 points), and of whole grains (0-3 points) earned higher points. Greater consumption quartile of red and processed meat was reverse-scored (3-0 points). Due to irregular intake distribution, SSB subcomponent scoring used absolute cut points for both sexes. Together, highly processed foods (HPF), refined grains, and SSB scores contributed 3 to 0 points. The final score ranged from 0 to 12, with 12 representing the highest diet quality. Poor overall diet quality was defined as a diet score in the bottom sex-specific quartile (men: 0 to <4.50; women: 0 to <4.75). For component scores, the poor scores were a score of 0 (lowest sex-specific quartile) for each component of the overall score (defined as vegetables: men, ≤2.1 servings per day; women, ≤2.4 servings per day; fruit: men, ≤0.9 servings per day; women, ≤1.1 servings per day; whole grains: men, ≤0.7 servings per day; women, ≤0.7 servings per day; red and processed meat: men, >1.5 servings per day; women, >1.2 servings per day; HPF and refined grains: men, >39% kcal; women, >40% kcal; SSB: ≥3 servings per week for men and women).

### Exposure Variables

The National Institutes of Health definition of Health Disparate Populations, which covers key aspects of social determinants of health,^32^ guided our selection of variables for this analysis. At enrollment, participants were asked if they consider themselves to be Hispanic or Latina/o (no/yes). Participants self-reported race as: American Indian or Alaskan Native; Asian; Native Hawaiian, or Pacific Islander; Black or African American; White; and/or other. If 2 races were selected and 1 included White, participants were categorized as the other race. Race and ethnicity was categorized as American Indian or Alaskan Native; Asian, Native Hawaiian, or Pacific Islander (grouped together because of small numbers); Hispanic or Latino; non-Hispanic Black (hereafter, *Black*); non-Hispanic White (hereafter, *White*; reference group owing to higher numbers); and other (also included multiple racial and ethnic designations, each too small to examine separately). Hispanic participants who did not identify as White (3.5% of Hispanic participants) were classified as other. Other individual-level exposures included household income, education, and residence. Participants’ home addresses were geocoded by Texas A&M University GeoServices.^33^ Rural-urban commuting area (RUCA) codes, which classify US census tracts according to urbanization, daily commuting, and population density from 2010 census and 2006 to 2010 American Community Survey data,^34^ were categorized as metropolitan, micropolitan, small town, and rural. The US Department of Agriculture Food Access Research Atlas database was used to define residence in a food desert, defined as the nearest grocery being farther than 1 mile in metropolitan and micropolitan settings, and farther than 10 miles for rural settings.^35^

### Exclusions

Among 177 345 CPS-3 participants who completed the FFQ, additional exclusions included 7484 participants for poor FFQ reporting (defined elsewhere^31^), 457 participants for top 0.05% of food group scores, 10 223 participants for geocode match score less than 80, 178 participants missing RUCA code, 42 participants missing food desert status, 280 participants missing race and ethnicity data, 2745 participants missing income data, and 605 participants missing education data.

### Statistical Analysis

Logistic regression models were used to cross-sectionally examine factors associated with poor diet quality, defined as lowest sex-specific category. Model 1 adjusted for age, sex, and energy intake; model 2 additionally included all key exposures; and model 3 included other potential confounders (ie, BMI, alcohol intake, and a comorbidity score) selected using backward elimination. To avoid controlling for consequences of poor diet, model 2 was considered the main model.

Two-way interactions of all key exposures compared all combinations to a common referent group and evaluated statistical significance of the cross-product term using the Wald Test. Two-sided *P* < .05 was considered statistically significant. SAS statistical software version 9.4 (SAS Institute) was used for all statistical analyses. Data were analyzed from February to November 2021.

## Results

The final analytic cohort included 155 331 participants, with a mean (SD) age of 52 (9.7) years and 32 216 men (20.7%) and 123 115 (79.3%) women. There were 1408 American Indian or Alaskan Native individuals (0.9%); 2721 Asian, Native Hawaiian, or Pacific Islander individuals (1.8%); 3829 Black individuals (2.5%); 7967 Hispanic individuals (5.1%); and 138 166 White individuals (88.9%). Characteristics according to quartile of ACS diet score are provided in Table 1. Poorer diet quality was observed among participants who were younger, American Indian or Alaska Native or Black, had lower income, had lower educational attainment, lived in a nonmetropolitan area, and lived in a food desert (Table 1). Those with lower diet quality were more likely to have never married, to work full time, to have obesity, to report minimal exercise, to currently smoke, to drink less alcohol, and to have 2 or more chronic medical conditions (Table 1). In addition, they were less likely to cook at home and more likely to frequent fast-food and full-service restaurants.

**Table 1.** Participant Characteristics by Adherence to the 2020 American Cancer Society Dietary Guidelines Score Among Adults in the Cancer Prevention Study-3

Characteristic	Dietary score, No. (%) (N = 155 331)^a^			
	Quartile 1 (n = 37 366)	Quartile 2 (n = 36 567)	Quartile 3 (n = 40 659)	Quartile 4 (n = 40 739)
Age, mean (SD), y	49.5 (9.64)	51.6 (9.69)	52.7 (9.63)	53.9 (9.48)
BMI, mean (SD)	29.5 (6.94)	28.2 (6.26)	27.2 (5.91)	25.5 (5.09)
Recreational physical activity, mean (SD), MET-h/wk	9.2 (8.54)	10.3 (8.75)	10.9 (8.82)	11.9 (9.08)
Sex				
Men	7262 (19.4)	8624 (23.6)	7460 (18.3)	8870 (21.8)
Women	30 104 (80.6)	27 943 (76.4)	33 199 (81.7)	31 869 (78.2)
Race and ethnicity				
American Indian or Alaskan Native	387 (1.0)	325 (0.9)	370 (0.9)	326 (0.8)
Asian, Native Hawaiian, or Pacific Islander	471 (1.3)	619 (1.7)	732 (1.8)	899 (2.2)
Black	1056 (2.8)	867 (2.4)	964 (2.4)	942 (2.3)
Hispanic	1896 (5.1)	1827 (5.0)	2085 (5.1)	2159 (5.3)
White	33 300 (89.1)	32 650 (89.3)	36 187 (89.0)	36 029 (88.4)
Other^b^	256 (0.7)	279 (0.8)	321 (0.8)	384 (0.9)
Income, $				
<25 000	1868 (5.0)	1462 (4.0)	1472 (3.6)	1409 (3.5)
25 000 to <50 000	5396 (14.4)	4258 (11.6)	4327 (10.6)	3913 (9.6)
50 000 to <75 000	7519 (20.1)	6632 (18.1)	6969 (17.1)	6595 (16.2)
75 000 to <100 000	6981 (18.7)	6493 (17.8)	7170 (17.6)	6910 (17.0)
100 000 to <125 000	5992 (16.0)	5983 (16.4)	6668 (16.4)	6582 (16.2)
125 000 to <150 000	3420 (9.2)	3796 (10.4)	4315 (10.6)	4268 (10.5)
≥150 000	6190 (16.6)	7943 (21.7)	9738 (24.0)	11 062 (27.2)
Educational level				
≤High school	4152 (11.1)	2953 (8.1)	2443 (6.0)	1689 (4.1)
Some college or 2-y degree	12 393 (33.2)	10 492 (28.7)	10 415 (25.6)	8079 (19.8)
College graduate	12 381 (33.1)	12 773 (34.9)	14 496 (35.7)	14 349 (35.2)
Graduate degree	8440 (22.6)	10 349 (28.3)	13 305 (32.7)	16 622 (40.8)
Rural-urban commuting area				
Metropolitan	30 387 (81.3)	31 360 (85.8)	35 810 (88.1)	36 967 (90.7)
Micropolitan	4593 (12.3)	3496 (9.6)	3211 (7.9)	2613 (6.4)
Small town	1478 (4.0)	1050 (2.9)	1033 (2.5)	728 (1.8)
Rural	908 (2.4)	661 (1.8)	605 (1.5)	431 (1.1)
Residing in a food desert				
No	34 010 (91)	33 917 (92.8)	38 051 (93.6)	38 374 (94.2)
Yes	3356 (9.0)	2650 (7.2)	2608 (6.4)	2365 (5.8)
Marital status				
Married or living with partner	28 174 (76.3)	28 024 (77.6)	31 251 (77.7)	31 358 (77.9)
Never married	3170 (8.6)	2655 (7.4)	2798 (7.0)	2848 (7.1)
Divorced, separated, or widowed	5588 (15.1)	5425 (15.0)	6184 (15.4)	6038 (15.0)
Work status				
Full time	27 469 (74.8)	25 371 (70.5)	26 540 (66.6)	24 840 (62.3)
Part time	3545 (9.7)	3901 (10.8)	5125 (12.9)	5934 (14.9)
Retired	3087 (8.4)	4153 (11.5)	5264 (13.2)	6146 (15.4)
Other	2600 (7.1)	2546 (7.1)	2942 (7.4)	2939 (7.4)
Smoking status				
Never	25 791 (69.2)	25 102 (68.9)	27 904 (68.8)	28 298 (69.7)
Current	1952 (5.2)	1123 (3.1)	873 (2.2)	492 (1.2)
Former	9509 (25.5)	10 213 (28.0)	11 754 (29.0)	11 816 (29.1)
Alcohol intake, drinks/d				
None	13 132 (35.2)	10 047 (27.5)	9957 (24.5)	9426 (23.2)
>0-1	18 944 (50.8)	19 268 (52.8)	21 845 (53.8)	21 592 (53.1)
>1	5240 (14.0)	7202 (19.7)	8783 (21.6)	9667 (23.8)
Family history of cancer				
No	14 047 (38.9)	13 195 (37.3)	14 345 (36.3)	14 060 (35.6)
Yes	22 091 (61.1)	22 216 (62.7)	25 141 (63.7)	25 481 (64.4)
Comorbidities^c^				
None	19 777 (52.9)	19 402 (53.1)	22 476 (55.3)	23 834 (58.5)
1	10 477 (28.0)	10 253 (28.0)	11 278 (27.7)	11 115 (27.3)
≥2	7112 (19.0)	6912 (18.9)	6905 (17.0)	5790 (14.2)
Full-service restaurant				
None or rarely	6797 (18.4)	6442 (17.8)	7288 (18.1)	8296 (20.6)
1 time/mo to 2 times/wk	27 546 (74.4)	27 231 (75.1)	30 538 (75.8)	29 792 (74.0)
≥3 times/wk	2675 (7.2)	2580 (7.1)	2458 (6.1)	2183 (5.4)
Fast food consumption				
None or rarely	9166 (24.6)	13 814 (38.0)	19 921 (49.3)	27 025 (66.8)
1 time/mo to 2 times/wk	20 789 (55.9)	18 725 (51.5)	17 971 (44.4)	12 408 (30.6)
≥3 times/wk	7231 (19.4)	3838 (10.6)	2538 (6.3)	1053 (2.6)
Homecooked meals				
None or rarely	1819 (4.9)	1173 (3.2)	844 (2.1)	552 (1.4)
1 time/mo to 2 times/wk	10 259 (27.6)	7481 (20.6)	6268 (15.5)	3877 (9.6)
≥3 times/wk	25 059 (67.5)	27 708 (76.2)	33 297 (82.4)	36 077 (89.1)

Abbreviations: BMI, body mass index (calculated as weight in kilograms divided by height in meters squared); MET, metabolic equivalent of task.

^a^ Total scores range from 0 to 12, with higher score indicating better diet quality. Quartiles are calculated using sex-specific cut points (men: quartile 1: 0 to <4.50; quartile 2: 4.50 to <6.25; quartile 3: 6.25 to <7.75; quartile 4: 7.75 to 12; women: quartile 1: 0 to <4.75; quartile 2: 4.75 to <6.25; quartile 3: 6.25 to <8.00; quartile 4: 8.00 to 12).

^b^ Includes individuals who identified as more than 1 minority race or ethnicity.

^c^ Comorbidities include diabetes; high blood pressure; high cholesterol; cancer; myocardial infarction; heart bypass surgery, angioplasty, or stent; stroke; and emphysema, chronic bronchitis, or chronic obstructive pulmonary disease.

All key exposures were statistically significantly associated with overall poor diet quality (Table 2; eFigures 1-5 in the Supplement). Adjusting for age, sex, and energy intake (model 1), American Indian and Alaska Native participants had 18% (95% CI, 5%-33%) higher odds of poor overall diet quality, and Black participants had 14% (95% CI, 6%-23%) higher odds of poor overall diet quality, compared with White participants. However, with mutual adjustment for other key exposures (model 2), the association for American Indian and Alaska Native participants was no longer statistically significant (eFigure 1 in the Supplement). American Indian and Alaska Native participants had higher odds of poor scores for red and processed meats and SSBs. Black participants had higher risk of poor vegetable and SSB scores, and lower risk of a poor HPF and refined grains score (ie, better concordance with recommendations) compared with White participants. In models including BMI, alcohol, and comorbidity score (model 3), Black race and ethnicity was no longer associated with poor diet quality. Study participants who identified as Asian, Native Hawaiian, and Pacific Islander; Hispanic/Latino; and as other race and ethnicity had significantly lower odds of poor diet quality, compared with White participants. Asian, Native Hawaiian, and Pacific Islander participants had lower odds of poor component scores (except for whole grains) compared with White participants (Table 2).

**Table 2.** Socioeconomic and Geographic Factors Associated With Poor Diet Quality Overall and Poor Diet Quality Component Scores^a^

Factor	Outcome, OR (95% CI)								
	Poor overall diet quality			Poor individual diet component score^b^					
	Model 1^c^	Model 2^d^	Model 3^e^	Vegetables	Fruits	Whole grains	RP meats	SSB	HPF/RG
Race and ethnicity									
American Indian or Alaska Native	1.18 (1.05-1.33)	1.04 (0.92-1.18)	0.96 (0.85-1.09)	0.88 (0.76-1.03)	1.12 (0.98-1.29)	0.99 (0.87-1.13)	1.18 (1.04-1.34)	1.21 (1.07-1.38)	0.78 (0.69-0.89)
Asian, Native Hawaiian, or Pacific Islander	0.54 (0.49-0.60)	0.67 (0.60-0.74)	0.69 (0.62-0.77)	0.56 (0.50-0.64)	0.77 (0.69-0.86)	1.19 (1.08-1.30)	0.66 (0.60-0.74)	0.67 (0.59-0.75)	0.83 (0.76-0.91)
Black	1.14 (1.06-1.23)	1.16 (1.08-1.25)	0.96 (0.89-1.03)	1.10 (1.01-1.20)	1.09 (1.00-1.18)	0.92 (0.85-1.00)	1.03 (0.95-1.12)	2.30 (2.14-2.48)	0.58 (0.53-0.63)
Hispanic	0.87 (0.82-0.92)	0.84 (0.79-0.88)	0.81 (0.76-0.85)	0.97 (0.91-1.03)	0.87 (0.82-0.93)	0.91 (0.86-0.97)	0.92 (0.87-0.98)	1.00 (0.94-1.06)	0.74 (0.70-0.79)
White	1 [Reference]	1 [Reference]	1 [Reference]	1 [Reference]	1 [Reference]	1 [Reference]	1 [Reference]	1 [Reference]	1 [Reference]
Other^f^	0.71 (0.62-0.82)	0.71 (0.61-0.81)	0.66 (0.57-0.76)	0.72 (0.61-0.85)	0.84 (0.72-0.99)	1.03 (0.89-1.18)	0.76 (0.66-0.88)	1.03 (0.89-1.18)	0.55 (0.48-0.64)
Income, $									
<25 000	1.25 (1.17-1.33)	1.08 (1.02-1.15)	1.01 (0.94-1.07)	1.27 (1.18-1.37)	1.26 (1.17-1.35)	1.06 (0.99-1.14)	0.88 (0.83-0.95)	1.28 (1.19-1.36)	0.93 (0.87-0.99)
25 000 to <50 000	1.20 (1.15-1.26)	1.10 (1.05-1.15)	1.06 (1.02-1.11)	1.14 (1.08-1.20)	1.14 (1.09-1.20)	1.03 (0.99-1.08)	1.01 (0.96-1.05)	1.17 (1.12-1.23)	1.02 (0.98-1.07)
50 000 to <75 000	1 [Reference]	1 [Reference]	1 [Reference]	1 [Reference]	1 [Reference]	1 [Reference]	1 [Reference]	1 [Reference]	1 [Reference]
75 000 to <100 000	0.89 (0.85-0.92)	0.95 (0.92-0.99)	0.98 (0.94-1.02)	0.91 (0.87-0.96)	0.91 (0.87-0.95)	1.02 (0.98-1.06)	1.00 (0.96-1.04)	0.85 (0.81-0.88)	0.94 (0.90-0.98)
100 000 to <125 000	0.78 (0.75-0.81)	0.89 (0.86-0.93)	0.96 (0.92-1.00)	0.86 (0.82-0.90)	0.85 (0.81-0.89)	0.98 (0.94-1.02)	1.00 (0.96-1.05)	0.77 (0.73-0.80)	0.94 (0.90-0.97)
125 000 to <150 000	0.69 (0.65-0.72)	0.83 (0.79-0.87)	0.92 (0.87-0.97)	0.74 (0.70-0.78)	0.81 (0.77-0.86)	1.03 (0.98-1.08)	0.97 (0.92-1.02)	0.69 (0.65-0.73)	0.89 (0.85-0.93)
≥150 000	0.54 (0.52-0.56)	0.71 (0.68-0.74)	0.84 (0.80-0.87)	0.69 (0.65-0.72)	0.77 (0.73-0.80)	1.03 (0.99-1.07)	0.86 (0.83-0.90)	0.55 (0.53-0.58)	0.78 (0.75-0.81)
Educational level									
≤High school	2.35 (2.25-2.46)	1.99 (1.90-2.09)	1.83 (1.75-1.92)	1.50 (1.42-1.58)	1.63 (1.55-1.72)	1.63 (1.55-1.71)	1.43 (1.36-1.50)	1.66 (1.58-1.75)	1.29 (1.23-1.35)
Some college or 2-y degree	1.57 (1.53-1.62)	1.42 (1.38-1.47)	1.32 (1.28-1.36)	1.11 (1.07-1.15)	1.23 (1.19-1.28)	1.33 (1.29-1.38)	1.33 (1.29-1.37)	1.36 (1.32-1.41)	1.08 (1.05-1.12)
College graduate	1 [Reference]	1 [Reference]	1 [Reference]	1 [Reference]	1 [Reference]	1 [Reference]	1 [Reference]	1 [Reference]	1 [Reference]
Graduate degree	0.72 (0.70-0.74)	0.76 (0.73-0.78)	0.77 (0.75-0.80)	0.84 (0.81-0.87)	0.84 (0.81-0.88)	0.88 (0.85-0.91)	0.76 (0.74-0.79)	0.82 (0.79-0.85)	0.97 (0.94-1.00)
RUCA									
Metropolitan	1 [Reference]	1 [Reference]	1 [Reference]	1 [Reference]	1 [Reference]	1 [Reference]	1 [Reference]	1 [Reference]	1 [Reference]
Micropolitan	1.76 (1.69-1.83)	1.50 (1.44-1.56)	1.43 (1.38-1.49)	1.21 (1.15-1.26)	1.18 (1.12-1.23)	1.01 (0.97-1.06)	1.46 (1.40-1.52)	1.37 (1.31-1.43)	1.20 (1.15-1.24)
Small town	1.89 (1.77-2.01)	1.54 (1.44-1.64)	1.48 (1.38-1.59)	1.22 (1.12-1.32)	1.14 (1.05-1.23)	1.03 (0.95-1.11)	1.64 (1.53-1.76)	1.28 (1.19-1.38)	1.12 (1.05-1.20)
Rural	1.99 (1.83-2.16)	1.61 (1.48-1.75)	1.55 (1.42-1.69)	1.34 (1.21-1.48)	1.19 (1.07-1.32)	1.00 (0.91-1.10)	1.80 (1.65-1.96)	1.19 (1.08-1.32)	1.12 (1.02-1.22)
Residing in a food desert									
No	1 [Reference]	1 [Reference]	1 [Reference]	1 [Reference]	1 [Reference]	1 [Reference]	1 [Reference]	1 [Reference]	1 [Reference]
Yes	1.43 (1.37-1.50)	1.17 (1.12-1.22)	1.12 (1.07-1.17)	1.05 (0.99-1.10)	1.08 (1.03-1.14)	1.08 (1.03-1.14)	1.14 (1.09-1.20)	1.17 (1.11-1.22)	0.99 (0.95-1.04)

Abbreviations: HPF/RG, highly processed foods and refined grains; RP, red and processed; RUCA, rural-urban commuting area; SSB, sugar sweetened beverages.

^a^ Total scores range from 0 to 12, with higher score indicating better diet quality. Poor overall diet quality is defined as a diet score in the bottom sex-specific quartile (men: 0 to <4.50; women: 0 to <4.75); poor component scores have a score of 0 (lowest sex-specific quartile) for servings per day (vegetables: men, ≤2.1; women, ≤2.4; fruit: men, ≤0.9; women, ≤1.1; whole grains: men, ≤0.7; women, ≤0.7; RP meat: men, >1.5; women, >1.2; HPF/RG: men, >39% kcal; women, >40% kcal). Poor SSB scores are defined as 3 or more servings per week in men and in women. Poor SSB scores were liberalized from the 7 or more servings per week cut point for the overall score (eTable 1 in the Supplement) to increase the percentage exposed to 26% for men and 16% for women, comparable to proportions for other components.

^b^ Model 2 (adjusted for age, sex, energy intake, and mutually adjusted for all main exposures) was used for the analyses by diet score components. Poor component scores represented less vegetables, fruits, whole grains and more RP meats, SSBs, and HPF/RG.

^c^ Adjusted for age, sex, and energy intake.

^d^ Mutually adjusted for all main exposures, age, sex, and energy intake.

^e^ Mutually adjusted for all main exposures, age, sex, energy intake, and additional covariates, including body mass index (calculated as weight in kilograms divided by height in meters squared and categorized as <25, 25 to <30, 30 to <35, ≥35, and missing), alcohol intake (none, >0 to 1 drink per day, >1-2 drinks per day, >2 drinks per day, and missing), and a comorbidity score (includes diabetes; high blood pressure; high cholesterol; cancer; myocardial infarction; heart bypass surgery, angioplasty, or stent; stroke; and emphysema, chronic bronchitis, or chronic obstructive pulmonary disease; 1 point for each; modeled as 0, 1, and ≥2).

^f^ Includes individuals who identified as more than 1 minority race or ethnicity.

Higher income and education were inversely and independently associated with risk of poor diet quality (Table 2; eFigure 2 and eFigure 3 in the Supplement). Each were each associated with better component scores, except income was not associated with higher whole grain scores. Participants in the lowest and highest income levels had higher (better) scores for red and processed meat recommendations than other income categories. Living in a nonmetropolitan area was associated with poorer diet quality, except for whole grains (Table 2; eFigure 4 in the Supplement), and residence in a food desert was independently associated with overall poor diet quality and poor diet components except HPF and refined grains (Table 2; eFigure 5 in the Supplement).

**Figure 1.**
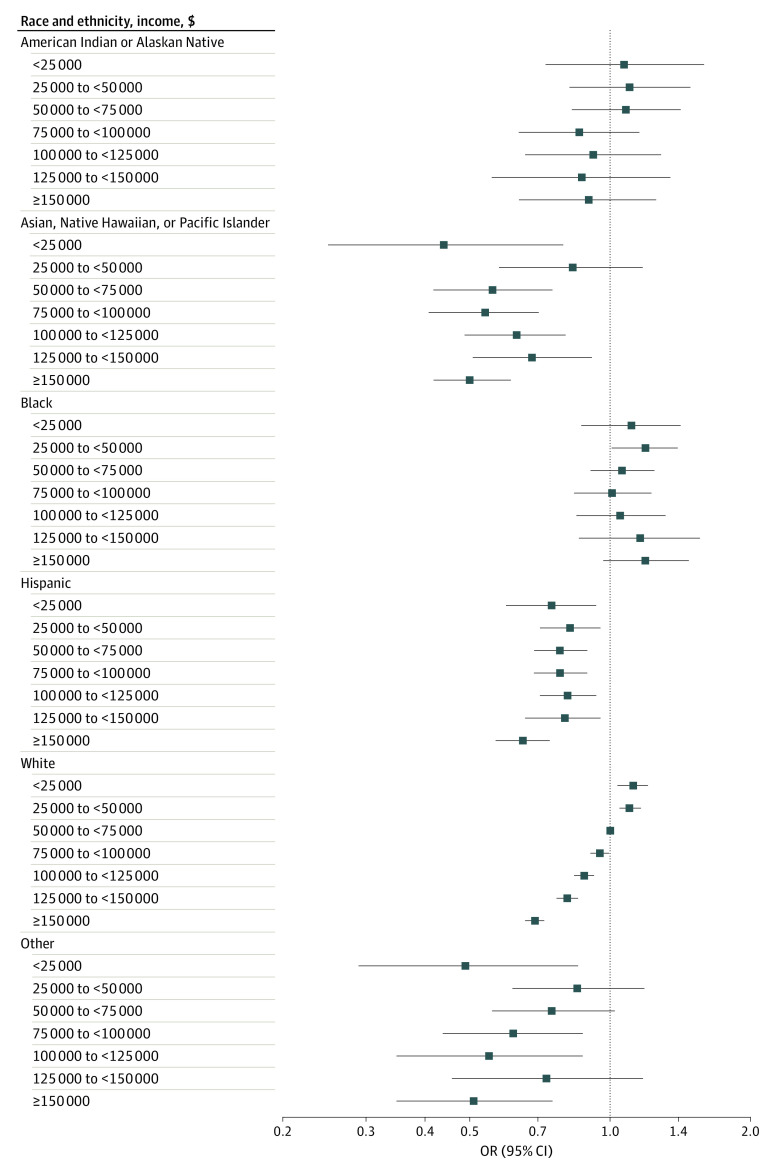
Association of Income Level and Risk of Poor Diet Quality, by Race and Ethnicity Odds ratio (OR) and 95% CI of lowest quartile of American Cancer Society diet quality score overall by income and stratified by race and ethnicity. Models included age, sex, energy intake, race and ethnicity, income, education, Rural-Urban Commuting Area code, and residence in a food desert, and a race × income interaction term. The reference group was White participants with income $50 000 to less than $75 000. *P* for interaction = .01.

**Figure 2.**
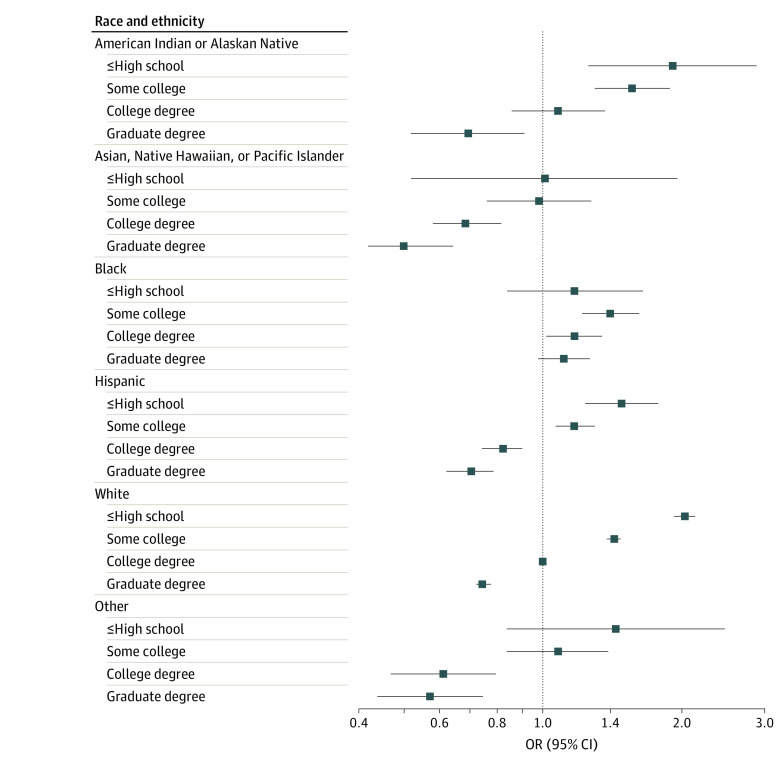
Association of Educational Attainment With Risk of Poor Diet Quality, by Race and Ethnicity Odds ratio (OR) and 95% CI of lowest quartile of American Cancer Society diet score overall, by attained education, stratified by race and ethnicity. Models included age, sex, energy intake, race and ethnicity, income, education, Rural-Urban Commuting Area code, and residence in a food desert, and a race × education interaction term. The Reference group was White participants with a college degree. *P* for interaction < .001.

Several significant interactions emerged across the exposures examined. Among White participants, higher income was incrementally associated with lower risk of poor diet quality (Figure 1; eTable 2 in the Supplement). However, compared with White participants earning $50 000 to less than $75 000, Black participants had 19% higher odds of poor diet quality at $25 000 to less than $50 000 per year (odds ratio, 1.19, 95% CI, 1.01-1.39) and diet quality was not higher with greater income. Asian, Native Hawaiian, or Pacific Islander individuals; Hispanic individuals; and individuals in the other racial and ethnic group had better diet quality at almost all income levels compared with the reference group. Educational attainment was associated with diet quality in all racial and ethnic groups, except for Black participants (Figure 2; eTable 2 in the Supplement). Greater educational attainment was also linearly associated with better diet quality in all rural and urban settings (eTable 3 and eFigure 6 in the Supplement). However, compared with college-educated participants residing in a metropolitan setting, individuals at all education levels in nonmetropolitan settings had higher risk of poor diet quality. Living in a food desert was associated with higher risk of poor diet quality in all 4 residential groups compared with individuals residing in a metropolitan setting who are not in a food desert; however, within micropolitan and rural locations, living in a food desert did not further increase risk (eTable 3 in the Supplement). No other statistically significant interactions were observed.

## Discussion

In this cross-sectional study of a demographically and geographically diverse cohort of 155 331 US adults, race and ethnicity, income, education, rural vs urban residence, and living in a food desert all were independently associated with poor diet quality. Compared with White participants, Black participants were at higher risk of poor diet quality and Asian, Native Hawaiian, and Pacific Islander and Hispanic/Latino participants were at lower risk in models mutually adjusted for socioeconomic and geographic factors. Higher income and education had protective associations against poor diet quality, but these associations were not the same across all racial and ethnic groups. In addition, while higher education was associated with better diet quality across the urban-rural spectrum, people with higher education living in nonmetropolitan areas still had lower diet quality than their counterparts living in metropolitan areas.

Disparities across poor dietary component scores are worth noting. Black participants had lower vegetable and fruit scores compared with White participants, consistent with national data showing Black persons having the lowest percentage meeting recommended vegetable intake (5.5% of Black persons; 10.5% of Hispanic/Latino persons; 9.5% of White persons).^22^ In this study, Black participants had 130% higher risk of a poor SSB score compared with White participants, while American Indian and Alaska Native adults had a 21% higher risk and Asian, Native Hawaiian, and Pacific Islander adults had a 33% lower risk. Data from NHANES^36^ show that the proportion of total beverage consumption as SSBs in the US is highest among Black (15%) and Hispanic/Latino (14%) adults, compared with Asian (4%) and White (9%) adults. While estimates of SSB intake in American Indian and Alaska Native adults are scant, a narrative review^37^ and NHIS data^38^ suggest high intakes in this group. Among other factors, SSBs are associated with weight gain, overweight, and obesity,^8^ and the prevalence of obesity in US adults is highest for non-Hispanic Black people, lowest for non-Hispanic Asian people, and similar among Hispanic/Latino and non-Hispanic White adults.^39^ White participants consumed more HPFs compared with other racial and ethnic groups, consistent with studies of shopping behaviors^40^ and data from NHANES.^41^ American Indian and Alaska Native participants in this study scored poorly for red and processed meats,^42^ which are associated with increased risk of several chronic diseases.^7,8,43,44,45^ American Indian and Alaska Native persons have higher rates of diabetes,^37^ obesity,^46^ and colorectal cancer^47^ than other groups, and are the only racial and ethnic group among whom mortality rates from colorectal cancer are not declining.

Gaps in diet quality by income and education have widened over the past decade in the US.^5^ Consistent with previous studies,^22,48,49,50,51,52,53,54,55^ our findings support an association of both income and education with consumption of a healthy diet, but this was not equal across racial and ethnic and geographic groups. Income was associated with diet quality only among White participants; it is possible that other groups have more resiliency (including likelihood to participate in subsidized food programs).^56^ However, compared with White participants, Black participants at all income and education levels had poorer diet quality. Poorer diet quality among Black participants at all income and education levels highlights the need to better understand factors that influence dietary intake in this population.

Multiple factors may shape diet quality among US Black persons, including cultural traditions, targeted advertising, and food availability, some regardless of income. Cultural eating practices may be influenced by historical trauma, limited access and resulting adaptation,^3,57^ and social aspects of eating.^58^ Prime time television shows oriented to Black people assessed in 1999 were 2-fold as likely to air food advertisements as general programming.^59^ Moreover, programming oriented to Black individuals featured more overweight characters and advertisements for SSBs and candy than general programming. Advertising is also targeted to local geographic areas based on demographic characteristics, such as racial and ethnic composition. In a study of 87 Designated Marketing Areas, each percentage increase in the proportion of the population that was Black was associated with 2.9 additional beverage advertisements and 2.2 additional food advertisements viewed.^60^ Advertisements of fast food, sweets, and SSBs were particularly prevalent in areas with larger proportions of Black residents with lower income. Furthermore, fewer supermarkets are found in Black vs White neighborhoods,^61,62^ while prevalence of fast-food restaurants is much higher.^63^ That risk of poorer diet quality in Black participants was attenuated with control for BMI, comorbidities, and alcohol likely reflects the correlates and/or consequences of dietary behaviors in this population.

The neighborhoods and built environments where people live are important social determinants of health.^64^ Foods available in rural areas tend to be less healthy, and healthy foods may be more expensive compared with that available in more urban areas.^65,66^ In the US, trends in diet quality were recently reported to vary by food source, with the retail grocery environment offering the best opportunity for improvements in diet quality.^67^ However, in rural areas, availability of grocery stores may be lower and availability of convenience stores higher.^68^ We found that individuals residing in all nonmetropolitan areas had a higher risk of poor diet quality than those in metropolitan areas. Although higher education was associated with mitigating that risk, it did not fully compensate for this increase. Finding solutions to improve diet quality among persons residing in food deserts has been challenging^69^; such initiatives require community- and system-level partnerships^70,71^ with attention to cultural relevance of healthy food options.^72^

Because poor diet quality is influenced by a complex range of interrelated factors, including structural factors, changes in health policies may be required to make a change.^73^ Health policy initiatives focusing on advertising, taxation, and pricing strategies to increase the competitiveness of more nutritionally dense foods and beverages could play a role in shifting diet quality. For example, SSB taxation is associated with successfully reducing SSB intake in the United Kingdom^74^ and in other countries, regions, and cities,^75^ although progress is lagging in the US.

### Limitations

This study has some limitations. Findings from this study may not be generalizable to the US population, as CPS-3 had a comparatively higher proportion of women and participants had relatively higher income and educational attainment and less racial and ethnic diversity. However, this large, nationwide cohort included sufficient numbers to examine associations of individual-level geographic and socioeconomic factors simultaneously. Furthermore, the direction of individual associations observed were generally consistent with nationally representative samples. This study included 66 participants who completed the Spanish-language questionnaire; as the diet validation study was conducted in English, we may have overestimated validity of the FFQ in Hispanic/Latino participants. The ideal score we created may not represent an ideal diet in all populations, and the FFQ was likely to lack some important foods in different subpopulations assessed, such as for Asian, Native Hawaiian, and Pacific Islander participants. An additional limitation to this research is the grouping together of heterogenous racial and ethnic groups to preserve statistical power, although variations within these groups are expected. Furthermore, since we categorized biracial White participants as the other race and ethnicity provided, dietary behaviors of these participants may not resonate fully with those of participants who self-identify as primarily American Indian or Alaska Native; Asian, Native Hawaiian, or Pacific Islander; Black; or Hispanic/Latino.

## Conclusions

In this cross-sectional study, we found multiple independent factors associated with diet quality in the CPS-3 cohort, including novel interactions among these factors. The ACS Guideline on Diet and Physical Activity for Cancer Prevention,^7^ provides dietary and lifestyle recommendations to reduce the cancer burden across all populations. These^7^ and other recommendations^76^ emphasize the need to examine the social, economic, and environmental contexts that may determine our dietary and lifestyle patterns, which also drive existing health disparities in the US. Further research to understand underlying social constructs driving diet quality, as well as potential barriers to consumption of healthier foods among individuals at risk of poor diet quality, is needed. These factors could be targeted for improved messaging, behavioral interventions, programs, and policies for everyone to have an equal opportunity to eat a healthy diet.

## Notes

Supplement. **eTable 1.** 2020 American Cancer Society Diet Guideline Score **eFigure 1.** Association of Race and Ethnicity With Risk of Poor Diet Quality Overall and Poor Diet Component Scores **eFigure 2.** Association of Income With Risk of Poor Diet Quality Overall and Poor Diet Component Scores **eFigure 3.** Association of Education With Risk of Poor Diet Quality Overall and Poor Diet Component Scores **eFigure 4.** Association of RUCA Code With Risk of Poor Diet Quality Overall and Poor Diet Component Scores **eFigure 5.** Association of Residence in a Food Desert and Risk of Poor Diet Quality Overall and Poor Diet Component Scores **eTable 2.** Social and Demographic Factors Associated With Poor Diet Quality by Race and Ethnicity **eTable 3.** Social and Demographic Factors Associated With Poor Diet Quality by Rural-Urban Commuting Area **eFigure 6.** Association of Educational Attainment With Risk of Poor Diet Quality, by RUCA
