# Supplementary material for: Association of Socioeconomic and Geographic Factors With Diet Quality in US Adults
Source: JAMA Netw Open. 2022 Jun 9;5(6):e2216406. doi: 10.1001/jamanetworkopen.2022.16406 (PMC9185183; doi:10.1001/jamanetworkopen.2022.16406)
Supplement: Supplement. — eTable 1. 2020 American Cancer Society Diet Guideline Score eFigure 1. Association of Race and Ethnicity With Risk of Poor Diet Quality Overall and Poor Diet Component Scores eFigure 2. Association of Income With Risk of Poor Diet Quality Overall and Poor Diet Component Scores eFigure 3. Association of Education With Risk of Poor Diet Quality Overall and Poor Diet Component Scores eFigure 4. Association of RUCA Code With Risk of Poor Diet Quality Overall and Poor Diet Component Scores eFigure 5. Association of Residence in a Food Desert and Risk of Poor Diet Quality Overall and Poor Diet Component Scores eTable 2. Social and Demographic Factors Associated With Poor Diet Quality by Race and Ethnicity eTable 3. Social and Demographic Factors Associated With Poor Diet Quality by Rural-Urban Commuting Area eFigure 6. Association of Educational Attainment With Risk of Poor Diet Quality, by RUCA [file jamanetwopen-e2216406-s001.pdf]

# Supplemental Online Content

McCullough ML, Chantaprasopsuk S, Islami F, et al. Association of socioeconomic and geographic factors with diet quality in US adults. *JAMA Netw Open*. 2022;5(6):e2216406. doi:10.1001/jamanetworkopen.2022.16406

**eTable 1.** 2020 American Cancer Society Diet Guideline Score

**eFigure 1.** Association of Race and Ethnicity With Risk of Poor Diet Quality Overall and Poor Diet Component Scores

**eFigure 2.** Association of Income With Risk of Poor Diet Quality Overall and Poor Diet Component Scores

**eFigure 3.** Association of Education With Risk of Poor Diet Quality Overall and Poor Diet Component Scores

**eFigure 4.** Association of RUCA Code With Risk of Poor Diet Quality Overall and Poor Diet Component Scores

**eFigure 5.** Association of Residence in a Food Desert and Risk of Poor Diet Quality Overall and Poor Diet Component Scores

**eTable 2.** Social and Demographic Factors Associated With Poor Diet Quality by Race and Ethnicity

**eTable 3.** Social and Demographic Factors Associated With Poor Diet Quality by Rural-Urban Commuting Area

**eFigure 6.** Association of Educational Attainment With Risk of Poor Diet Quality, by RUCA

This supplemental material has been provided by the authors to give readers additional information about their work.

© 2022 McCullough ML et al. *JAMA Network Open.*

**eTable 1.** 2020 American Cancer Society Diet Guideline Score[a]

| Sub score category | Score | Criteria for low score | Points | Criteria for highest score | Points |
|---|---|---|---|---|---|
| Fruit & Vegetables | 0-3 | | | | |
| Vegetable intake | | Lowest quartile | 0 | Highest quartile | .75 |
| | | ≤2.1 svgs/d in men | | >4.9 svgs/d in men | |
| | | ≤2.4 svgs/d in women | | >5.7 svgs/d in women | |
| Vegetable variety | | Lowest quartile | 0 | Highest quartile | .75 |
| | | ≤14 unique vegetables/mo in men | | >22 unique vegetables/mo in men | |
| | | ≤15 unique vegetables/mo in women | | >23 unique vegetables/mo in women | |
| Fruit intake | | Lowest quartile | 0 | Highest quartile | .75 |
| | | ≤0.9 svgs/d in men | | >2.6 svgs/d in men | |
| | | ≤1.1 svgs/d in women | | >2.9 svgs/d in women | |
| Fruit variety | | Lowest quartile | 0 | Highest quartile | .75 |
| | | ≤6 unique fruits/mo in men | | >12 unique fruits/mo in men | |
| | | ≤8 unique fruits/mo in women | | >13 unique fruits/mo in women | |
| Whole grains | 0-3 | Lowest quartile | 0 | Highest quartile | 3 |
| | | ≤0.7 svgs/d in men | | >1.9 svgs/d in men | |
| | | ≤0.7 svgs/d in women | | >1.7 svgs/d in women | |
| Red and processed meat | 0-3 | Highest quartile | 0 | Lowest quartile | 3 |
| | | >1.5 svgs/d in men | | ≤0.6 svgs/d in men | |
| | | >1.2 svgs/d in women | | ≤0.5 svgs/d in women | |
| Sugar-sweetened beverages and highly processed foods/refined grains | 0-3 | | | | |
| SSB intake | | SSB: ≥7 svgs/wk | 0 | SSB: None | 1.5 |
| HPF/RG intake | | HPF/RG, highest quartile | 0 | HPF/RG, lowest quartile | 1.5 |
| | | >39% kcal in men | | ≤25% kcal in men | |
| | | >40% kcal in women | | ≤24% kcal in women | |
| Total Score | 0-12 | | | | |

Abbreviations: svgs/d, servings per day; mo, month; svgs/wk, servings per week; SSB, sugar-sweetened beverages; HPF/RG, highly processed foods/refined grains

© 2022 McCullough ML et al. *JAMA Network Open.*

**eTable 1. 2020 American Cancer Society Diet Guideline Score, Continued**

[a] Components scored based on sex-specific distribution quartiles. Cutpoints for quartiles 2 and 3 are as follows for men and women respectively: vegetable intake: quartile 2: >2.1 to ≤3.2 and >2.4 to ≤3.7, quartile 3: >3.2 to ≤4.9 and >3.7 to ≤5.7; vegetable variety: quartile 2: >14 to ≤18 and >15 to ≤19, quartile 3: >18 to ≤22 and >19 to ≤23; fruit intake: quartile 2: >0.9 to ≤1.6 and >1.1 to ≤1.9, quartile 3: >1.6 to ≤2.6 and >1.9 to ≤2.9; fruit variety: quartile 2: >6 to ≤10 and >8 to ≤11, quartile 3: >10 to ≤12 and >11 to ≤13; whole grain intake: quartile 2: >0.7 to ≤1.3 and >0.7 to ≤1.1, quartile 3: >1.3 to ≤1.9 and >1.1 to ≤1.7; red and processed meat intake: quartile 2: >0.6 to ≤1.0 and >0.5 to ≤0.8, quartile 3: >1.0 to ≤1.5 and >0.8 to ≤1.2; highly processed foods/refined grains intake: quartile 2: >25% to ≤32% and >24% to ≤32%, quartile 3: >32% to ≤39% and >32% to ≤40%, except sugar-sweetened beverages which were categorized as none, >0 to <3 servings/week, ≥3 to <7/week, 7+ per week. Intermediate scores are 0.25 and 0.5, 1 and 2, and 0.5 and 1.0 for subscores ranging from 0 to 0.75, 0 to 3, and 0 to 1.5 respectively.

© 2022 McCullough ML et al. *JAMA Network Open.*

**eFigure 1.** Association of Race and Ethnicity With Risk of Poor Diet Quality Overall and Poor Diet Component Scores

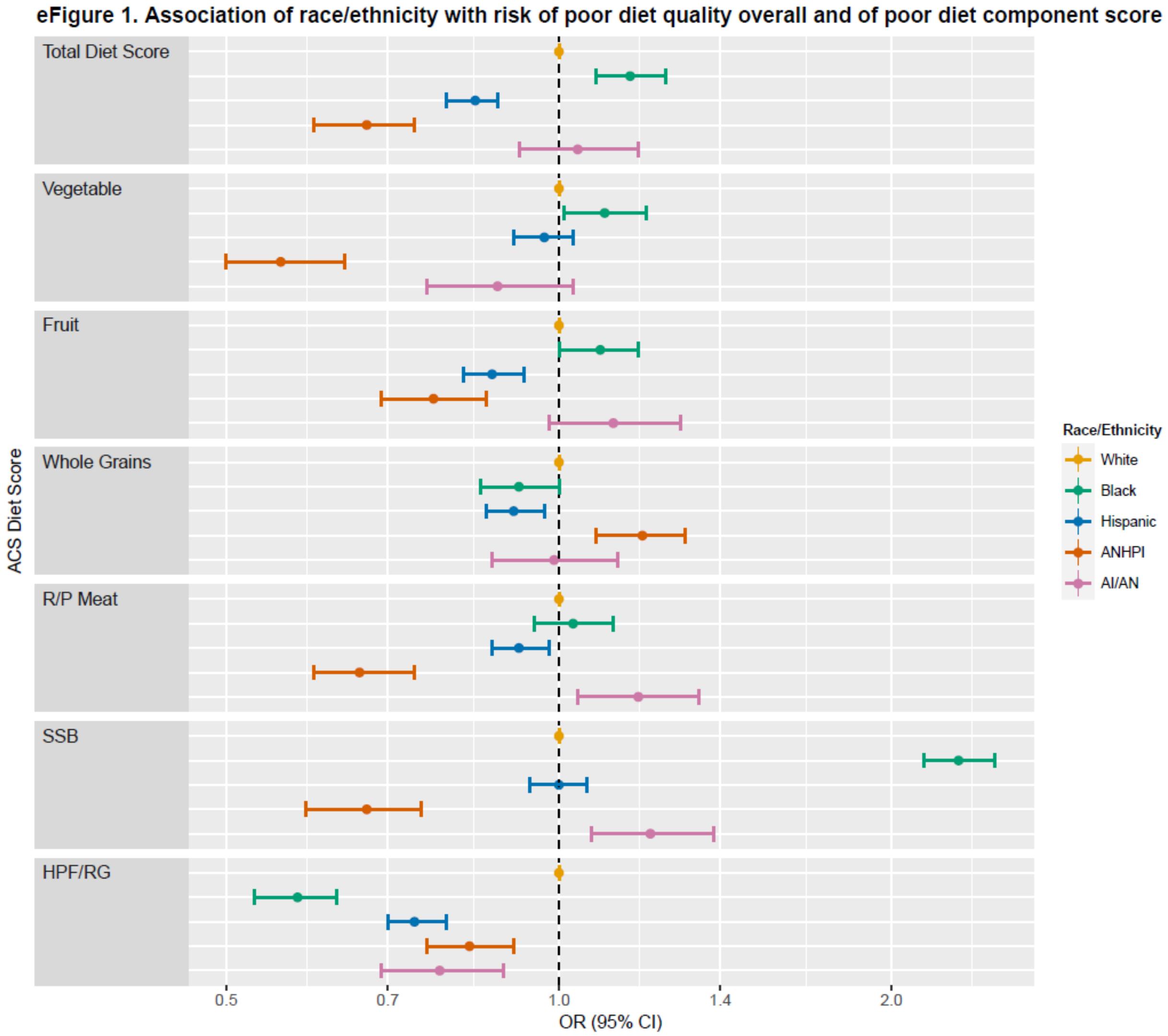


Odds ratio (OR) and 95% confidence interval (95% CI) of lowest quartile of ACS diet score, overall, or of low diet component score (see Table 2 footnotes), according to race/ethnicity (referent group: White). Models included age, sex, energy intake, income, education, RUCA code and residence in a food desert (Model 2). AI/AN=American Indian/Alaska Native; ACS=American Cancer Society; ANHPI=Asian/Native Hawaiian/Pacific Islander.

© 2022 McCullough ML et al. *JAMA Network Open.*

**eFigure 2.** Association of Income With Risk of Poor Diet Quality Overall and Poor Diet Component Scores

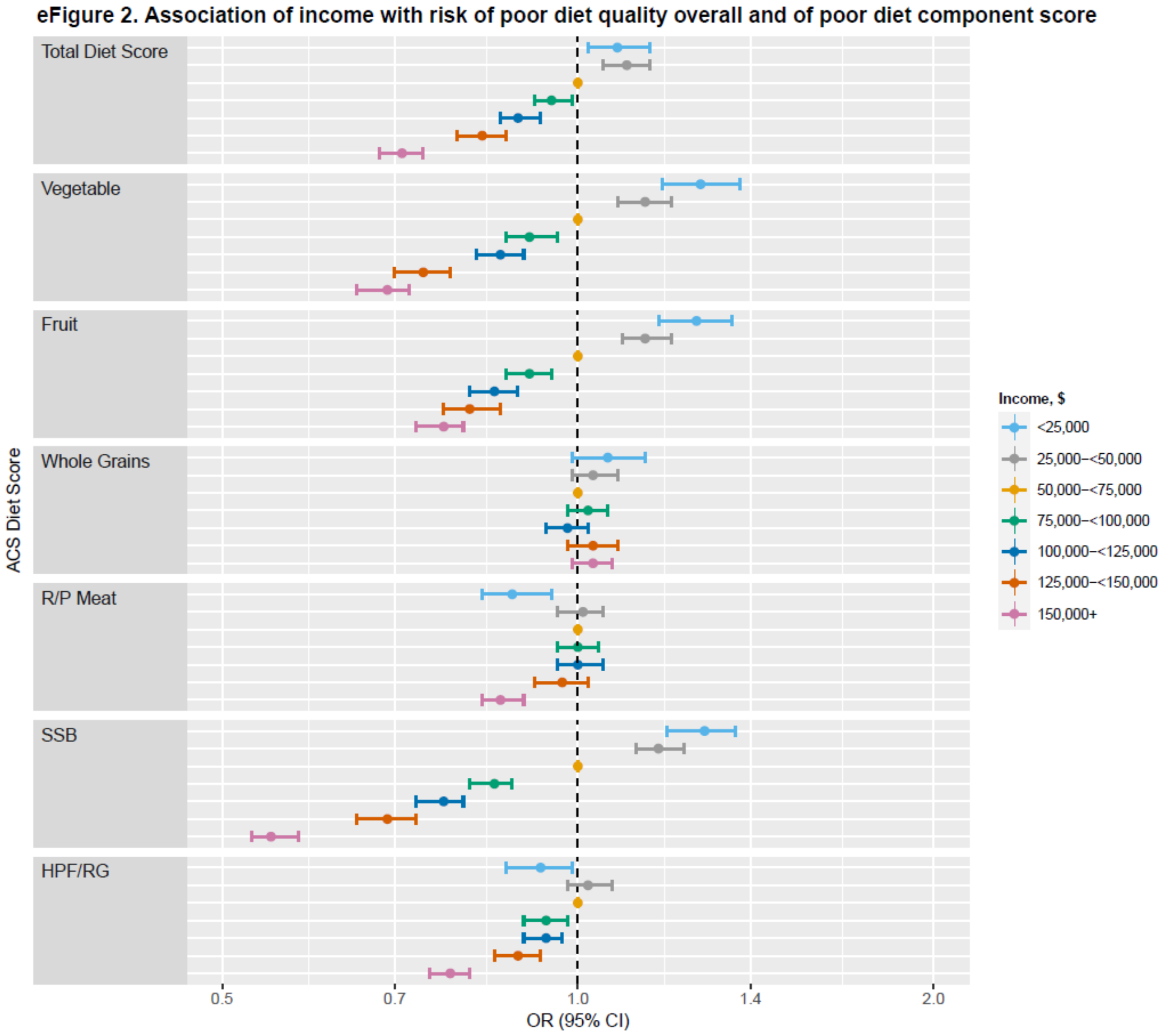


Odds ratio (OR) and 95% confidence interval (95% CI) of lowest quartile of ACS diet score, overall, or of low diet component score (see Table 2 footnotes), according to income (referent group: $50,000-<75,000). Models included age, sex, energy intake, race/ethnicity, education, RUCA code and residence in a food desert (Model 2).

© 2022 McCullough ML et al. *JAMA Network Open.*

**eFigure 3.** Association of Education With Risk of Poor Diet Quality Overall and Poor Diet Component Scores

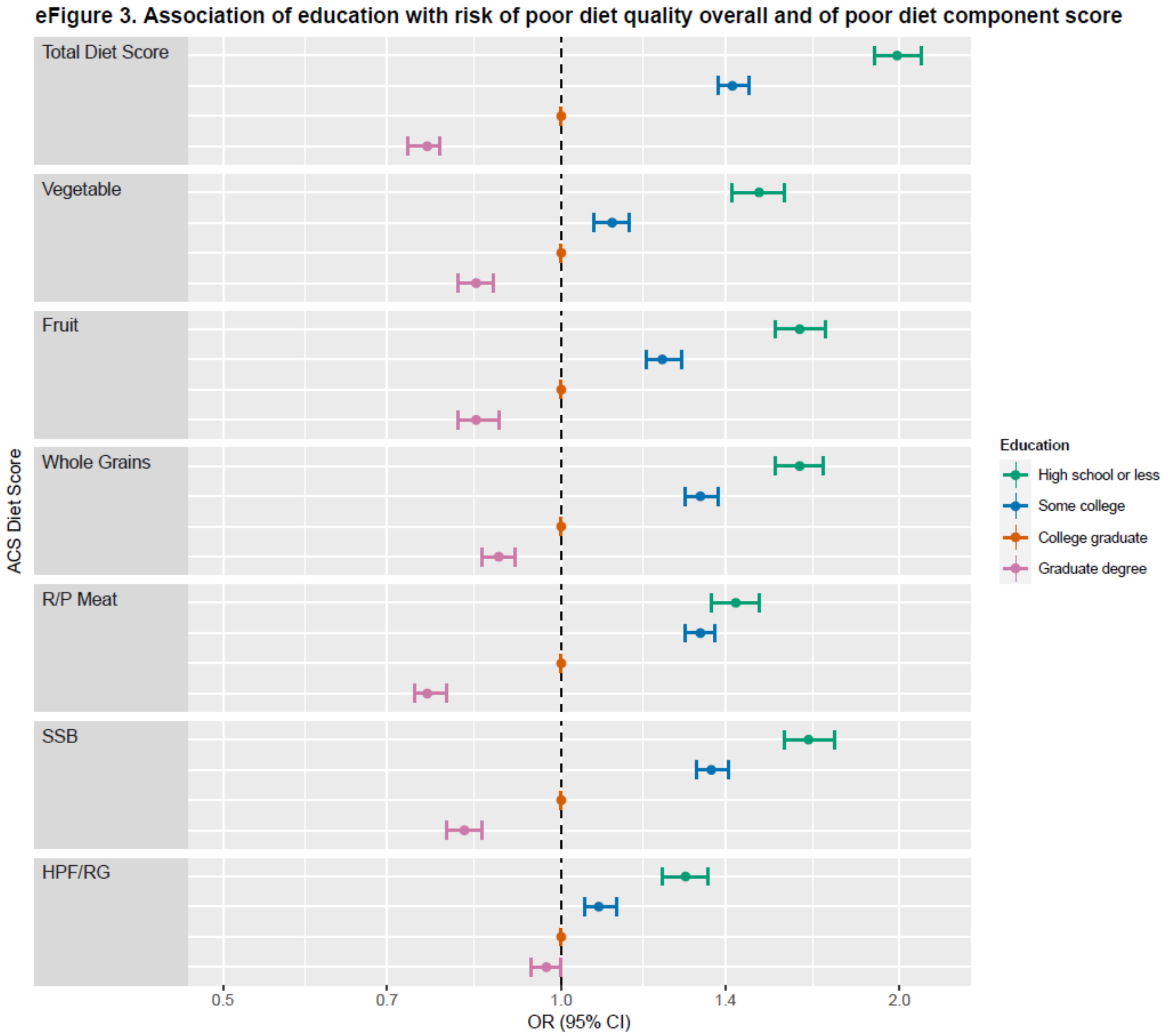


Odds ratio (OR) and 95% confidence interval (95% CI) of lowest quartile of ACS diet score, overall, or of low diet component score (see Table 2 footnotes), according to education (referent group: college graduate). Models included age, sex, energy intake, race/ethnicity, income, RUCA code and residence in a food desert (Model 2).

© 2022 McCullough ML et al. *JAMA Network Open.*

**eFigure 4.** Association of RUCA Code With Risk of Poor Diet Quality Overall and Poor Diet Component Scores

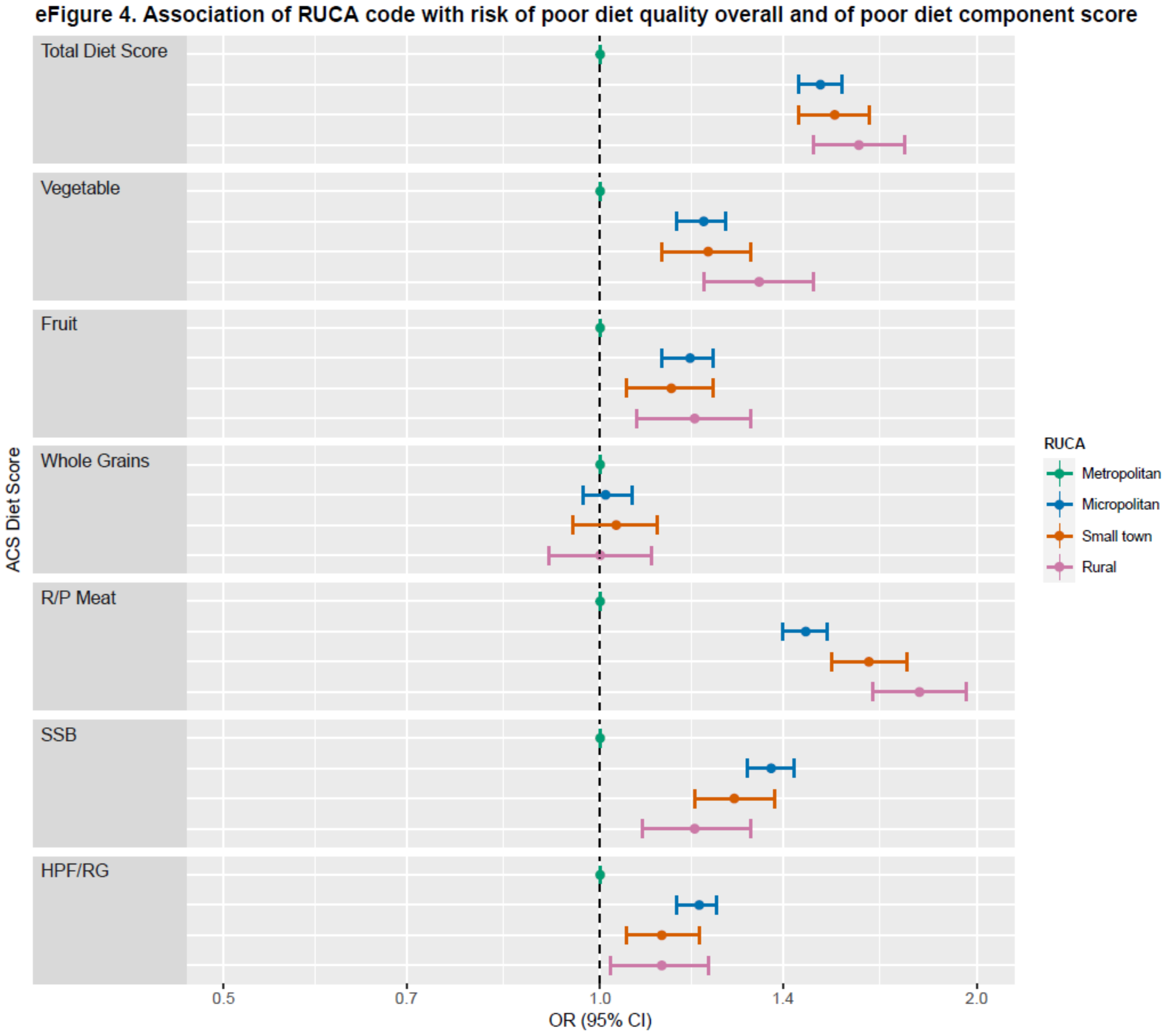


Odds ratio (OR) and 95% confidence interval (95% CI) of lowest quartile of ACS diet score, overall, or of low diet component score (see Table 2 footnotes), according to RUCA code (Rural Urban Commuting Area code, referent group: metropolitan). Models included age, sex, energy intake, race/ethnicity, income, education, and residence in a food desert (Model 2).

© 2022 McCullough ML et al. *JAMA Network Open.*

**eFigure 5.** Association of Residence in a Food Desert and Risk of Poor Diet Quality Overall and Poor Diet Component Scores

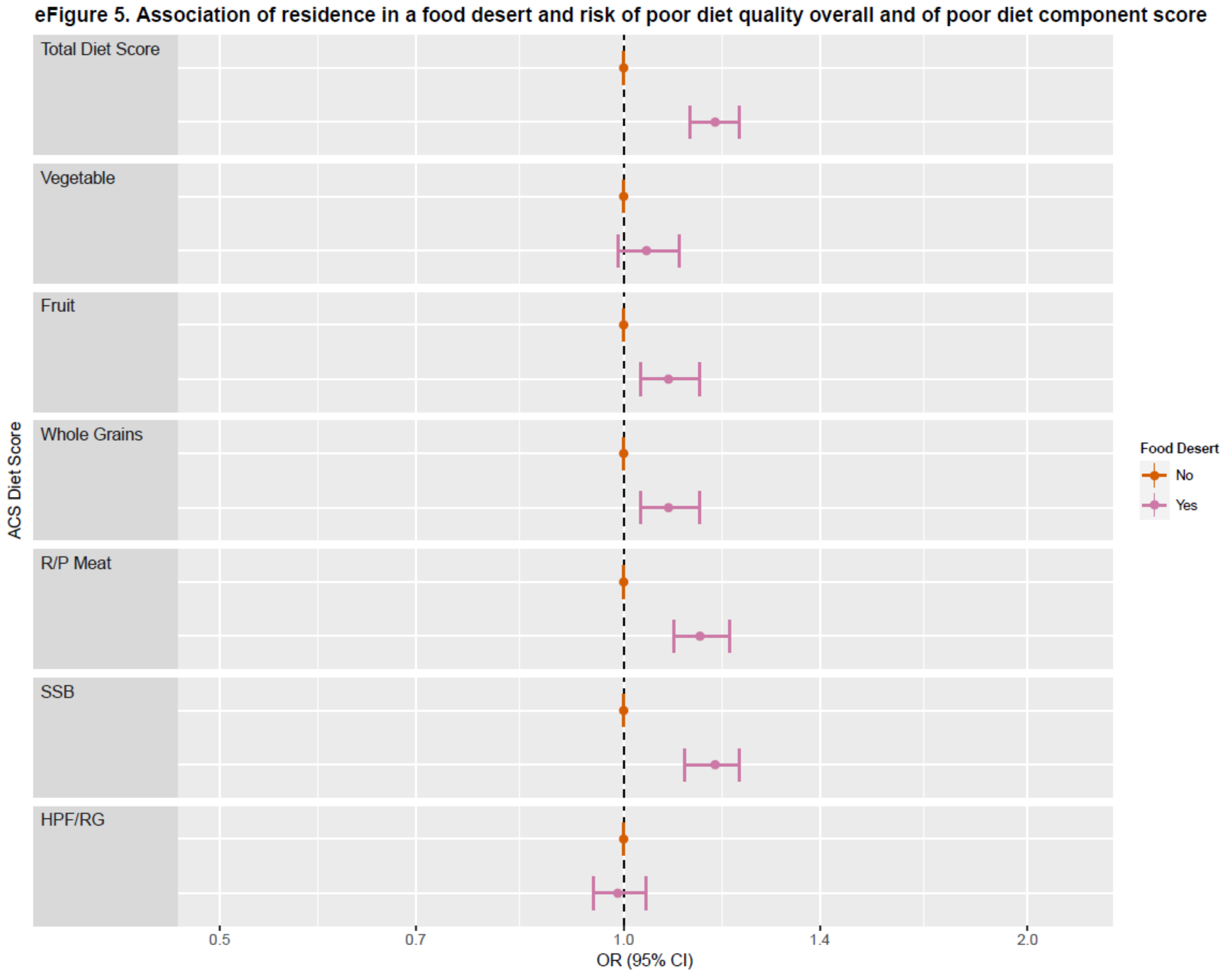


Odds ratio (OR) and 95% confidence interval (95% CI) of lowest quartile of ACS diet score, overall, or of low diet component score (see Table 2 footnotes), according to residence in a food desert (referent group: no). Models included age, sex, energy intake, race/ethnicity, income, education and RUCA code (Model 2).

© 2022 McCullough ML et al. *JAMA Network Open.*

**eTable 2.** Social and Demographic Factors Associated With Poor Diet Quality by Race and Ethnicity[a]

| | Race/ethnicity | | | | | | |
|---|---|---|---|---|---|---|---|
| | White | Black | Hispanic | ANHPI | AI/AN | Other | p-int |
| **RUCA**[b] | | | | | | | 0.01 |
| Metropolitan | 1.00 (Ref) | 1.15 (1.07-1.25) | 0.82 (0.77-0.87) | 0.67 (0.60-0.74) | 1.07 (0.93-1.22) | 0.73 (0.63-0.84) | |
| Non-metropolitan | 1.51 (1.46-1.56) | 1.87 (1.38-2.54) | 1.52 (1.30-1.78) | 1.09 (0.65-1.81) | 1.43 (1.09-1.88) | 0.75 (0.44-1.27) | |
| **Income, $**[c] | | | | | | | 0.01 |
| <25,000 | 1.12 (1.04-1.20) | 1.11 (0.87-1.41) | 0.75 (0.60-0.93) | 0.44 (0.25-0.79) | 1.07 (0.73-1.58) | 0.49 (0.29-0.85) | |
| 25,000-<50,000 | 1.10 (1.05-1.16) | 1.19 (1.01-1.39) | 0.82 (0.71-0.95) | 0.83 (0.58-1.17) | 1.10 (0.82-1.48) | 0.85 (0.62-1.18) | |
| 50,000-<75,000 | 1.00 (Ref) | 1.06 (0.91-1.24) | 0.78 (0.69-0.89) | 0.56 (0.42-0.75) | 1.08 (0.83-1.41) | 0.75 (0.56-1.02) | |
| 75,000-<100,000 | 0.95 (0.91-0.99) | 1.01 (0.84-1.22) | 0.78 (0.69-0.89) | 0.54 (0.41-0.70) | 0.86 (0.64-1.15) | 0.62 (0.44-0.87) | |
| 100,000-<125,000 | 0.88 (0.84-0.92) | 1.05 (0.85-1.31) | 0.81 (0.71-0.93) | 0.63 (0.49-0.80) | 0.92 (0.66-1.28) | 0.55 (0.35-0.87) | |
| 125,000-<150,000 | 0.81 (0.77-0.85) | 1.16 (0.86-1.55) | 0.80 (0.66-0.95) | 0.68 (0.51-0.91) | 0.87 (0.56-1.34) | 0.73 (0.46-1.17) | |
| 150,000+ | 0.69 (0.66-0.72) | 1.19 (0.97-1.47) | 0.65 (0.57-0.74) | 0.50 (0.42-0.61) | 0.90 (0.64-1.25) | 0.51 (0.35-0.75) | |
| **Education**[d] | | | | | | | <0.0001 |
| High school or less | 2.03 (1.93-2.13) | 1.17 (0.84-1.64) | 1.48 (1.24-1.77) | 1.01 (0.52-1.95) | 1.91 (1.26-2.89) | 1.44 (0.84-2.47) | |
| Some college/2-year degree | 1.43 (1.38-1.47) | 1.40 (1.22-1.61) | 1.17 (1.07-1.29) | 0.98 (0.76-1.27) | 1.56 (1.30-1.88) | 1.08 (0.84-1.38) | |
| College graduate | 1.00 (Ref) | 1.17 (1.02-1.34) | 0.82 (0.74-0.90) | 0.68 (0.58-0.81) | 1.08 (0.86-1.36) | 0.61 (0.47-0.79) | |
| Graduate degree | 0.74 (0.72-0.77) | 1.11 (0.98-1.26) | 0.70 (0.62-0.78) | 0.50 (0.42-0.58) | 0.69 (0.52-0.91) | 0.57 (0.44-0.74) | |

Abbreviations: ANHPI, Asian/Native Hawaiian, Pacific Islander; AI/AN, American Indian/Alaskan Native; RUCA, rural-urban commuting area

[a] For exposures with statistically significant interactions. Poor diet quality is defined as a diet score in the bottom 25% sex-specific quartile
[b] Models included age, sex, energy intake, race/ethnicity, income, education, RUCA code and residence in a food desert, and a race*RUCA interaction term
[c] Models included age, sex, energy intake, race/ethnicity, income, education, RUCA code and residence in a food desert, and a race*income interaction term
[d] Models included age, sex, energy intake, race/ethnicity, income, education, RUCA code and residence in a food desert, and a race*education interaction term

© 2022 McCullough ML et al. *JAMA Network Open.*

**eTable 3.** Social and Demographic Factors Associated With Poor Diet Quality by Rural-Urban Commuting Area[a]

| | Rural-Urban Commuting Area | | | | |
|---|---|---|---|---|---|
| | Metropolitan | Micropolitan | Small town | Rural | p-int |
| **Education**[b] | | | | | 0.03 |
| High school or less | 2.05 (1.94-2.16) | 2.90 (2.61-3.21) | 2.83 (2.40-3.32) | 2.54 (2.07-3.10) | |
| Some college/2-year degree | 1.42 (1.38-1.47) | 2.15 (2.01-2.29) | 2.26 (2.03-2.52) | 2.18 (1.90-2.49) | |
| College graduate | 1.00 (Ref) | 1.47 (1.36-1.58) | 1.50 (1.32-1.71) | 2.01 (1.71-2.36) | |
| Graduate degree | 0.75 (0.73-0.78) | 1.18 (1.08-1.29) | 1.23 (1.05-1.44) | 1.25 (1.01-1.56) | |
| **Residing in a food desert**[c] | | | | | 0.02 |
| No | 1.00 (Ref) | 1.53 (1.47-1.60) | 1.53 (1.42-1.65) | 1.65 (1.50-1.80) | |
| Yes | 1.22 (1.15-1.28) | 1.58 (1.44-1.73) | 1.89 (1.59-2.25) | 1.62 (1.25-2.08) | |

[a] For exposures with statistically significant interactions. Poor diet quality is defined as a diet score in the bottom 25% sex-specific quartile

[b] Models included age, sex, energy intake, race/ethnicity, income, education, RUCA code and residence in a food desert, and a RUCA*education interaction term

[c] Models included age, sex, energy intake, race/ethnicity, income, education, RUCA code and residence in a food desert, and a RUCA*food desert interaction term

© 2022 McCullough ML et al. *JAMA Network Open.*

**eFigure 6.** Association of Educational Attainment With Risk of Poor Diet Quality, by RUCA

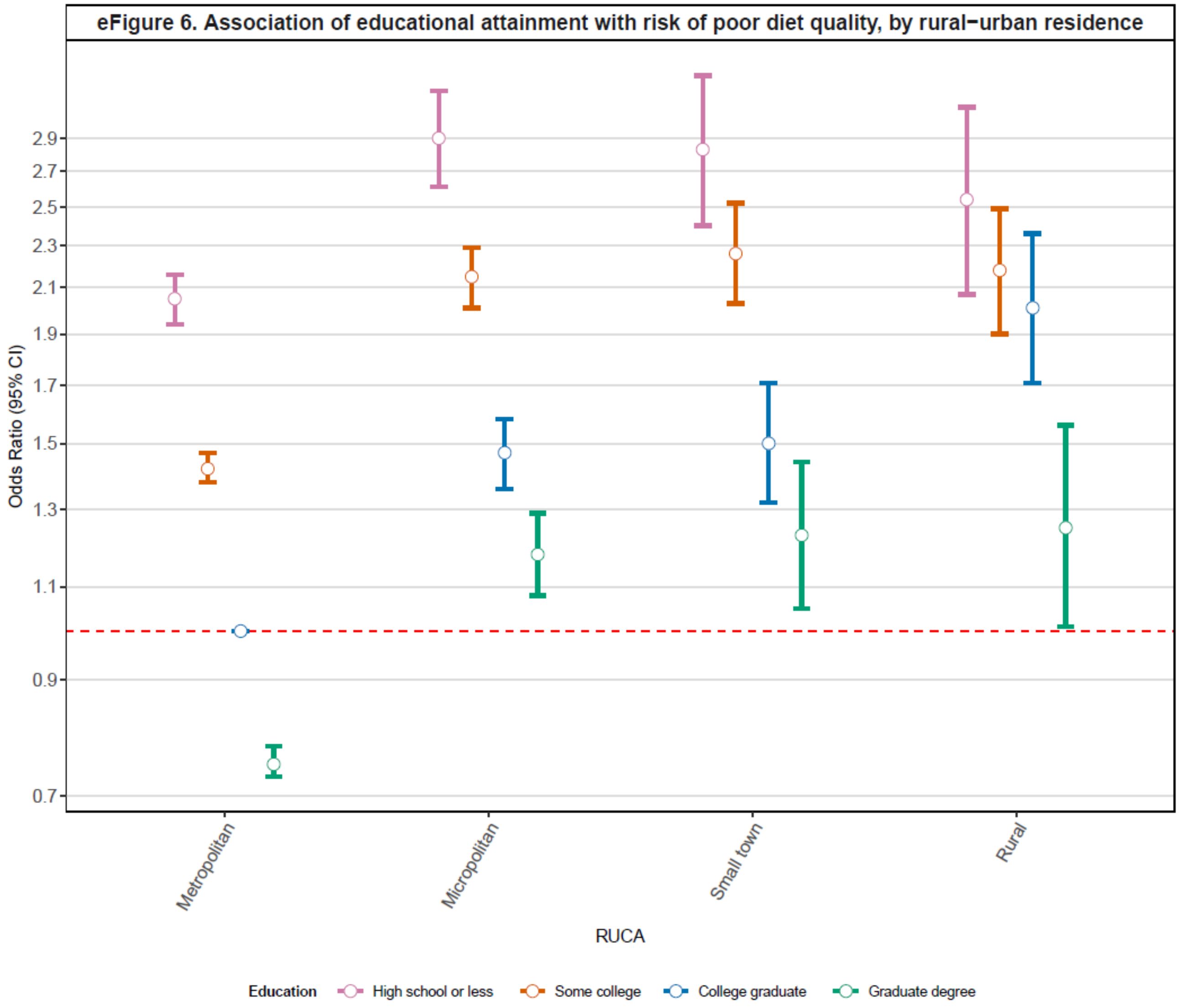


Odds ratio (OR) and 95% confidence interval (95% CI) of lowest quartile of ACS diet score overall by attained education, stratified by RUCA code (Rural Urban Commuting Area code) classification. Models included age, sex, energy intake, race/ethnicity, income, education, RUCA code and residence in a food desert, and a RUCA*education interaction term. Referent group: metropolitan/college degree). $P_{interaction}<0.03$

© 2022 McCullough ML et al. *JAMA Network Open.*
